# First molecular detection of *Toxoplasma gondii* in vegetable samples in China using qualitative, quantitative real-time PCR and multilocus genotyping

**DOI:** 10.1038/s41598-019-54073-6

**Published:** 2019-11-26

**Authors:** Anna Lass, Liqing Ma, Ioannis Kontogeorgos, Xueyong Zhang, Xiuping Li, Panagiotis Karanis

**Affiliations:** 1 Department of Tropical Parasitology, Institute of Maritime and Tropical Medicine in Gdynia, Medical University of Gdansk, 9b Powstania Styczniowego Str., 81-519 Gdynia, Poland; 2 State Key Laboratory of Plateau Ecology and Agriculture, Center for Biomedicine and Infectious Disease, Qinghai University, 1#Wei’er Road, Qinghai Biological Scientific Estate Garden, Xining, 810016 P.R. China; 3 Marine Sciences Department, School of Environment, University of the Aegean, University Hill, 88 100 Mytilene, Greece; 4 Cologne University, Medical Faculty and University Hospital, Cologne, Germany; 5 University of Nicosia, Medical School, 46 Makedonitissas Avenue, CY-2417, P.O. Box 24005, CY-1700 Nicosia, Cyprus

**Keywords:** Parasitology, Risk factors

## Abstract

*Toxoplasma gondii* infection is becoming increasing problem in China but there is no data concerning contamination of vegetables intended for consumption with this parasite. The aim of the present study was to investigate fresh vegetables originated from open markets located in the Xining City, the Qinghai-Tibet Plateau (QTP), P.R. China for their contamination with *T. gondii*. A total of 279 fresh vegetable samples were collected and analysed using real-time PCR assay targeting B1 gene and multilocus genotyping. *T. gondii* DNA was found in 10 (3.6%) samples tested; eight of them represented *T. gondii* type I and remaining two *T. gondii* type II. The approximate level of contamination of positive vegetables samples, estimated based on quantitative real-time PCR (qPCR), ranged between less than one and 27000 *T. gondii* oocysts per sample, with majority not exceeding several oocysts per sample. The results of the study confirmed that *T. gondii* is present in vegetables offered in open markets in the Qinghai province, P.R. China; eating them unwashed and raw may therefore pose a threat to consumers. This is the first investigation describing *T. gondii* detection in fresh vegetables intended for consumption collected from the territory of P.R. China using sensitive molecular tools.

## Introduction

*Toxoplasma gondii* is a cosmopolitan protozoan parasite able to infect humans and warm-blooded animals. Toxoplasmosis is one of the most prevalent parasitic infections in humans^1–3^. The disease is generally asymptomatic in immunocompetent individuals, it may take, however, a severe course up to life-threatening conditions in immunodeficient patients^4^, as well as in immature foetuses and infants, if the mother suffered from primary infection during pregnancy^5,6^.

There are two main routes of acquiring *T. gondii* infection: consumption of raw and undercooked meat of infected animals containing cysts filled with parasites or accidental ingestion of oocysts excreted to the environment with faeces of infected Felidae, being the only definitive hosts of the parasite^7–11^. Presence of *T. gondii* has been confirmed in water, soil, and air in different parts of the world, for example in Poland^12–14^, Germany^15^, France^16,17^, Scotland^18^, Brazil^19,20^, Ecuador^21^, Iran^22,23^ and Turkey^24^. Oocysts may also persist on the surface of fruit and vegetables; experiments have demonstrated that they may stay viable on raspberries stored at 4 °C for eight weeks^25^. Subsequent environmental studies confirmed presence of *T. gondii* in fresh fruit and vegetables samples indicating them as potential source of infection in humans^26–29^.

There are three major genetic lineages of *T. gondii* (I, II, III) defined, frequently observed in Europe and North America^30^ and a rising number of atypical genotypes present in different parts of the world^31–34^. The most common genotype of *Toxoplasma* found in Asia is genotype ToxoDB#9 (Chinese 1) with vast majority isolates detected in China^35–38^, but also found in Sri Lanka and Vietnam^35,39^. Type I is mostly present in eastern parts of Asia including South Korea, eastern provinces of China, peninsular Malaysia and Myanmar^35^. Less frequent in Asia are genotypes ToxoDB#1 and #3 (type II). Predominating in Europe, *T. gondii* type II is present in western and central Asia including Turkey, Qatar, Iran and the western provinces of China, which suggests a continuum between type II and Chinese 1 in the Eurasian continent^35^. In-depth sequencing studies suggest the same ancestral origin for type II and Chinese 1^40–42^ and studies on migration pathways for *Toxoplasma* also support the hypothesis that Chinese 1 preceded type II^35^. Genotypes ToxoDB#2 (type III) together with atypical genotypes are the least prevalent in China^35^. Genotyping of *Toxoplasma* isolates coming from environmental samples has not been performed in China yet.

Toxoplasmosis in China is a consistent problem, despite being lower^43^ than the commonly accepted world average^1,44^; according to nationwide studies involving several thousands of examined humans, the national prevalence of toxoplasmosis fluctuated from 5.9% in the early ‘90 s^45^ to 7.9% in the ‘00 s^46^. A more recent nationwide study involving 2,008,561 women before pregnancy reported even lower prevalence (2.6%)^47^, depicting a decreasing trend in younger generations in rural areas. Similar trend has been identified in the nationwide survey as well^46^, where the highest prevalence regards citizens over 80 years old. There are also differences in the prevalence of the disease between different ethnic groups^46,48^, which are merely reflecting the differences in the exposure to the parasite relevant to occupational and societal (rural – urban residence) conditions and eating habits.

Regarding detection of *Toxoplasma* in the food chain, the majority of studies is focused on commercially raised livestock such as sheep^49,50^, goats^51,52^, bovines^53,54^, swine^55,56^ and poultry^57,58^. Attention has been given, recently, into commercially available meat as well^59–64^. This is important since traditional eating habits in some regions of China include consumption of raw or undercooked meat^65^, which has been associated along with domestic hygiene measures and consumption of raw milk with increased infection rates in China^48,66–68^ and in other countries^69,70^. Nevertheless, the foodborne route of toxoplasmosis through meat consumption has been well documented both globally and in China. The risk resulting from attachment of oocysts in vegetables and fruits, however, has not been studied extensively. In China particularly, we did not find any investigation regarding occurrence of *Toxoplasma* in fresh vegetables or fruits. Presence of *T. gondii* has been confirmed so far only in soil samples in different regions of the country, including north-western (Qinghai and Gansu), central (Hubei), north-eastern (Heilongjiang) and eastern (Jiangsu) provinces^71–74^ which demonstrates contamination of the environment with this parasite, and makes contamination of fresh fruit and vegetables likely.

The aim of the present study was to estimate the possible occurrence of *T. gondii* in vegetables available for consumers in open markets in Xining City, Qinghai province, P.R. China as well as to determine oocysts charge (number of dispersive forms of parasite) of investigated samples and genotype of obtained *T. gondii* isolates via sensitive molecular tools (real-time PCR, qPCR, multilocus PCR/RFPL analysis).

## Samples and Study Area

**Figure 1:**
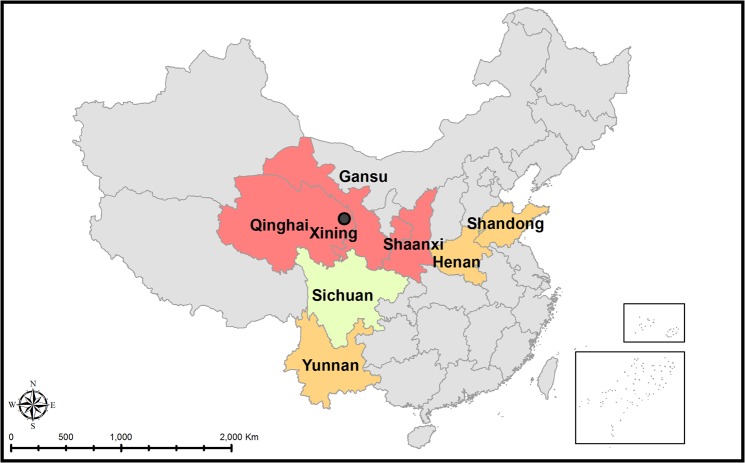
Location of sampling place (open markets) - Xining City, Qinghai Province, P.R. China and provinces where investigated vegetables grow. Colours represent occurrence of particular genotypes in Chinese provinces^35^: pink refers to provinces where type II is prevalent; yellow refers to provinces where Chinese 1 genotype is prevalent and type I is common; olive represents province with scarce data about prevalence of *T. gondii* genotypes.

A total of 279 fresh vegetable samples were collected between January and August 2016 from open markets located in the Xining City, Qinghai Province, western China (Fig. 1). Following vegetables were collected and examined: lettuce (*Lactuca sativa*) (n = 71), spinach (*Spinacia oleracea*) (n = 50), pak choi (*Brassica rapa* subsp. *chinensis*) (n = 34), Chinese cabbage (*Brassica pekinensis*) (n = 26), rape (*Brassica napus*) (n = 22), Asparagus (*Asparagus officinalis*) (n = 18), *Chrysanthemum coronarium* (n = 16), endive (*Cichorium endivia*) (n = 14), Chinese chives (*Allium tuberosum*) (n = 11), cabbage (*Brassica oleracea* L. var. *capitata*) (n = 9), red cabbage (*Brassica oleracea*) (n = 8). The samples were placed in disposable bags and transported to the laboratory.

### Origin of vegetables

Vegetables available at open markets in Xining City are taken from the Qinghai-Tibet Plateau Agricultural Products Distribution Centre, a big hub for agricultural products. Only about 10% of them are grown in the Qinghai Province and the vast majority are transported there from other provinces including: Sichuan, Shandong, Shaanxi, Henan, Yunnan and Gansu provinces (Fig. 1). We were not able, however, to identify the origin of each separate vegetable that was sampled. Vegetables are cultured in greenhouses and farmlands which are located in the suburbs of the cities or in rural areas close to countryside. These places may attract animals including cats being definitive hosts of *T. gondii* and contamination of plants with oocysts is probable.

## Results

**Figure 2:**
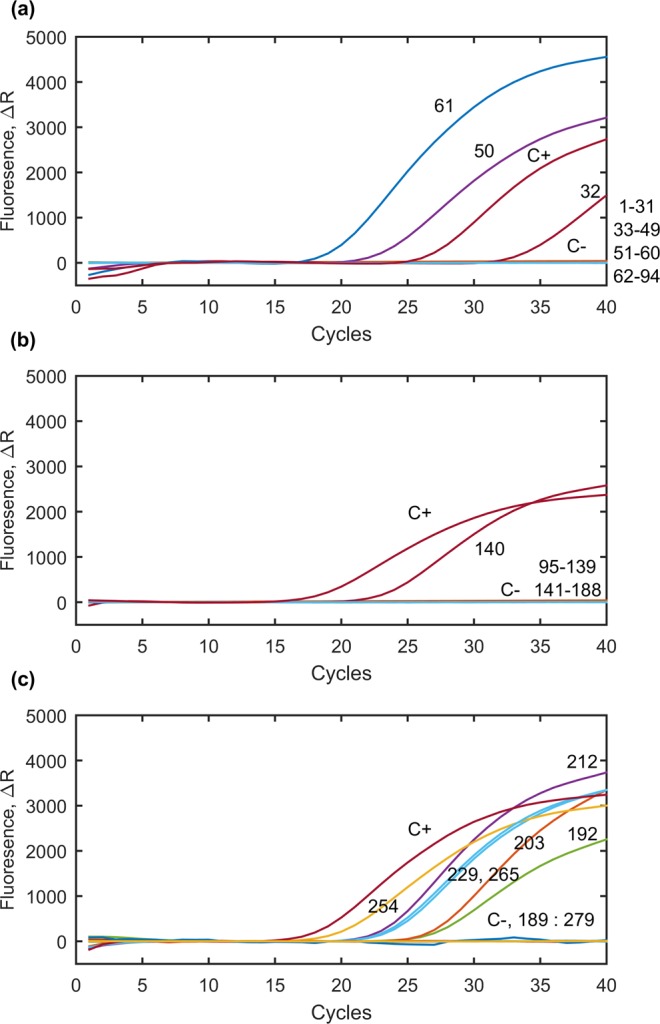
Results of *T. gondii* DNA detection in vegetable samples collected from the area of Xining City, Qinghai Province, P.R. China using real-time PCR. C+ refers to positive control; C- refers to negative control; numbers of lines correspond to numbers of templates as follows 32, 50, 61, 140, 192, 203, 212, 229, 254, 265 positive samples and 1–31, 33–49,51–60, 62–94, 95–139, 141–188, 189–191, 193–202, 204–211, 213–228, 230–253, 255–264, 267–279 negative samples.

In total, 279 fresh vegetable samples originated from open markets in the Xining City, QTP, P.R. China were examined with real-time PCR assays based on the *T. gondii* B1 gene. The presence of *T. gondii* DNA was recorded in 10 (3.6%) samples investigated, including lettuce, spinach, Chinese cabbage (pak choi), red cabbage and rape samples (Table 1, Fig. 2). Sequencing of positive samples and comparison with the *T. gondii* sequence deposited in the GenBank displayed that the obtained nested PCR products were fragments of the *T. gondii* B1 gene (Table 2). Samples representing remaining types of vegetables were negative for *Toxoplasma* DNA (Table 1). The IPC test excluded presence of PCR inhibition in investigated samples that could influence the results (details are available in Supplementary Table S3).

**Table 1:** Summary results of the detection of *Toxoplasma gondii* DNA in vegetable samples collected from open markets located in Xining City, Qinghai Province, P.R. China using real-time PCR.

Type of the sample	Samples investigated with real-time PCR									
	January		March		July		August		Total samples	
	No.	(%)	No.	(%)	No.	(%)	No.	(%)	No.	(%)
Lettuce (*Lactuca sativa*)	0/6	—	0/2	—	3/48	(6.2)	2/15	(13.3)	5/71	(7.0)
Spinach (*Spinacia oleracea*)	0/15	—	0/0	—	2/27	(7.4)	0/8	—	2/50	(4.0)
Pak choi (*Brassica rapa* subsp. *Chinensis*)	1/15	(6.7)	0/9	—	0/10	—	0/0	—	1/34	(2.9)
Chinese cabbage (*Brassica pekinensis*)	0/12	—	0/13	—	0/1	—	0/0	—	0/26	—
Rape (*Brassica napus*)	1/12	(8.3)	0/0	—	0/3	—	0/7	—	1/22	(4.5)
Asparagus (*Asparagus officinalis*)	0/14	—	0/4	—	0/0	—	0/0	—	0/18	—
*Chrysanthemum coronarium*	0/9	—	0/1	—	0/6	—	0/0	—	0/16	—
Endive (*Cichorium endivia*)	0/0	—	0/0	—	0/8	—	0/6	—	0/14	—
Chinese chives (*Allium tuberosum*)	0/9	—	0/1	—	0/0	—	0/1	—	0/11	—
Cabbage (*Brassica oleracea* L. var. *capitata*)	0/6	—	0/3	—	0/0	—	0/0	—	0/9	—
Red cabbage (*Brassica oleracea*)	1/2	(0.5)	0/2	—	0/1	—	0/3	—	1/8	(1.2)
Total results	3/100	(3.0)	0/35	(0.0)	5/104	(4.8)	2/40	(5.0)	10/279	(3.6)
Monthly/Total results	3/279	(1.0)	0/279	(0.0)	5/279	(1.8)	2/279	(0.7)

**Table 2:** Characteristics of positive vegetable samples collected from open markets in the Xining City, Qinghai Province, P.R. China, regarding detection (reproducibility of real-time PCR results), quantification, and genotyping of *T. gondii*.

Template No	Type of sample	Real-time PCR result	N	OCS	CCS	*T. gondii* genotype
32	Pak choi (*Brassica rapa* subsp. *chinensis*)	3/3	4.94e + 001	1.7	13.6	I
50	Red cabbage (*Brassica oleracea*)	3/3	6.99e + 002	25	200	II
61	Rape (*Brassica napus*)	3/3	7.74e + 005	27640	221120	II
140	Spinach (*Spinacia oleracea*)	3/3	9.56e + 004	340	2730	I
192	Lettuce (*Lactuca sativa*)	3/3	9.66e + 000	0.3	2.76	I
203	Lettuce (*Lactuca sativa*)	3/3	7.00e + 002	25	200	I
212	Lettuce (*Lactuca sativa*)	3/3	7.56e + 005	27000	432000	I
229	Spinach (*Spinacia oleracea*)	3/3	6.64e + 001	2.4	19.2	I
254	Lettuce (*Lactuca sativa*)	3/3	1.29e + 002	4.6	36.8	II
265	Lettuce (*Lactuca sativa*)	3/3	5.30e + 000	0.18	1.44	I

(3/3) three positive results obtained per three repeats of real-time PCR.

N – initial copy number (number of copies of *T. gondii* B1 gene) present in washings of investigated vegetable sample after flocculation, calculated using qPCR.

OCS – oocyst charge of the sample - equivalent of number of *T. gondii* oocysts which could be present in vegetable samples calculated based on initial copy number (N).

CCS - calculated equivalent of initial number of *T. gondii* cells (sporozoites) present in positive vegetable samples (OCS x 8, one oocysts consist of two sporocysts filled with four sporozoites each).

Genotyping of ten positive samples using set of ten different genetic markers (SAG1, SAG2, SAG3, BTUB, GRA6, c22-8, c29-2, L358, PK1, Apico) showed that the majority (eight) of obtained *Toxoplasma* isolates represent *T. gondii* genotype I. In case of the remaining two positive samples *T. gondii* genotype II was detected (Table 2).

Regarding sampling period, the majority of vegetable samples positive for *Toxoplasma* DNA were detected during summer months, 5/104 (4.8%) and 2/40 (5%) in July and August respectively (Table 1). Lower number of positive samples was noted during winter and early spring season. Presence of parasite was confirmed in only 3/100 (3%) of vegetable samples collected in January and none of the samples collected in March 0/35 (0%) (Table 1). Chi-square test, however didn’t show statistical dependence between number of positive samples and month χ^2^ = 2.08 (p = 0.55). Frequency of positive vegetable samples collected during particular sampling months against the total number of vegetable samples collected in this study (n = 279) was as follows: 5/279 (1.8%), 3/279 (1.1%) and 2/279 (0.7%) for July, January and August respectively (Table 1).

Results of quantitative real-time PCR enabled the calculation of equivalent of *T. gondii* oocysts present in positive vegetable samples, in the final suspension obtained after flocculation (Table 2). The approximate level of contamination of positive vegetables samples ranged between less than one and 27000 *T. gondii* oocysts per sample, with majority not exceeding several oocysts per sample (Table 2) (detailed information about qPCR including standard curve and positive samples is available in Supplementary Tables S1 and S2).

## Discussion

In the present study we successfully detected *Toxoplasma* DNA in fresh vegetable samples collected from local open markets located in the Xining City, the capital of Qinghai province in China. To the best of our knowledge, this is the first investigation describing *T. gondii* detection in vegetable samples collected from territory of P.R. China using sensitive molecular tools. *T. gondii* DNA was detected in ten out of 279 tested samples. The results of our study confirmed that fresh vegetables available in this part of China were contaminated with *T. gondii* and they may pose a potential threat for public safety, particularly, people consuming them raw and unwashed.

Contamination of vegetables goes hand in hand with contamination of surrounding environment, mainly soil and water, and the driving force behind it is felid activity. While there is an absence of studies regarding *T. gondii* presence in water, *Toxoplasma* has been detected in soil in different parts of China, both in urban^72,74,75^ and agricultural areas^71,73,74^. These studies included Qinghai and Gansu provinces in the north-western part, Harbin and Wuhan in the centre and Nanjing in the east and all of them showed over 10% of contamination rate, in some cases 30%^71–75^. Most probably stray cats play a pivotal role in epidemiology of toxoplasmosis; they roam freely and may void invasive oocysts of the parasite to the environment with faeces. It is possible that these animals may reach areas where fruit and vegetables are growing and contaminate it with the *Toxoplasma* oocysts. In China, epidemiological surveys have been conducted regarding prevalence of *T. gondii* in cats; a systematic review of publications between 1995 and 2016 included 38 studies and estimated the average infection rate in 20%, and seroprevalence in stray cats was significantly higher than in pet cats^76^. Investigations performed in north-western China in Lanzhou City, Gansu Province showed 21.3% examined cats infected including 15.6% and 45.2% for household and stray cats respectively^77^. In QTP, types of Felidae have also been speculated as a potential source of *T. gondii*, such as Pallas’ cat (*Otocolobus manul*) and Chinese mountain cat (*Felis bieti*), both species populating the plateau in high numbers^78^. Therefore, the contaminating agent and background contamination are well established theoretically. Data from QTP and other parts of China, including provinces from which vegetables are transported to the Qinghai Province, indicate presence of *Toxoplasma* in the environment.

Presence of *T. gondii* DNA has been confirmed in different type of vegetable samples investigated in this study including lettuce, spinach, pak choi, rape and red cabbage samples. We have noticed differences in number of positive vegetable samples between seasons in which they were collected. *Toxoplasma* DNA was more frequently found in samples collected during summer months in comparison to winter and early spring. Different factors may influence this situation, such as felid activity, water or aeolian erosion and agricultural practice. Vegetables may originate from greenhouses or farmlands; when they are grown on open fields they are exposed to the rain and wind that enable transportation of soil particles and oocysts from soil surface on the vegetables. The irrigation practice may provide a similar mechanism of transportation, from soil to vegetables, or become the contamination agent in cases where the irrigational water is contaminated. Moreover, open fields are accessible to animals including felids that may excrete the oocysts of *T. gondii* and therefore, we have found higher number of positive samples among collected during warm months when animal activity is rising and the use of greenhouses is reduced.

The level of contamination of collected samples in this study was 3.6%, which is lower than what has been previously reported in other countries^28,29^. These studies included collection of vegetables covering a spectrum of agricultural practices, logistics and distribution as well as environmental conditions. The study conducted in Paraná, Brazil, with a tropical climate, showed 3.8% of vegetable samples collected from sales outlets and production sites positive to *Toxoplasma*^29^. In the same area, vegetables sold in community fairs, producers’ market and generally open markets, from local producers with the corresponding logistics, and showed 9.5% samples contaminated^26^. The study which was conducted in northern Poland, an area with oceanic climate, included fruit and vegetables from open markets, from direct producers but also from greengrocers and supermarkets, while the products originated from the general area of sampling; the overall rate of contaminated samples was 9.7%^28^. On the other hand, a broad study in major Canadian cities, under completely different conditions, showed quite lower contamination level (0.26%)^27^; it regarded imported pre-packaged or bulk green vegetables from USA and Mexico, with the corresponding logistics. In the aforementioned study in Poland, samples taken from supermarkets, where washing and sanitizing methods may have been applied, showed no contamination^28^. Our study regards, on one hand vegetables supplied in open markets, but on the other they are transported hundreds of kilometres away from the cultivation site and provided through a centralised system to the small sellers in these markets, constituting a combination of the previous examples. Therefore they may have more sophisticated packaging than in the cases of direct produce or decentralised distribution, and possibly some form of washing or sanitizing for a part of them.

The primary contamination of vegetables during cultivation has been established in a number of cases^26,28^. It should be emphasized that while comparing data from cited studies, different factors that could influence the results should be taken under consideration such as limitations of recovery, concentration and detection methods used, as well as the environmental contamination background of the investigated region, we may also assume that the handling of vegetables after their collection influences the contamination level and the threat for the public. Nevertheless, a coordinated screening of soil, vegetables and the end product at the hands of the consumer should be organised to properly assess the risk at each level of production.

Genotyping of positive samples collected in this study indicated that *T. gondii* isolates represent type I (eight samples) and II (two samples). Although, *T. gondii* - Chinese 1 genotype is widely prevalent in China with a rising gradient from west to east, in the northwest of China where Qinghai is situated, the predominating genotypes are type II and I followed by Chinese 1^35^. Our results follow a similar pattern, since vegetables may originate from the north-western provinces, Qinghai, Gansu and Shaanxi, or Henan, Shandong and Yunnan, where *Toxoplasma* - type I genotype is common^35^ (Fig. 1). The study of agricultural soil contamination with *Toxoplasma* could help establish a hypothesis, but such studies in China have not included genotyping^71,73,74^. Hence, we assume that the localities where the particular vegetables were cultivated were contaminated with the aforementioned genotypes.

Additionally, an equivalent of the approximate oocyst charge using qPCR was established to a mean level of positive samples. The order of magnitude of the number of oocysts that might be present in positive samples ranged from one to 10^4^. For half of samples number of oocysts was below 10, and only two of them were highly contaminated. Actual contamination, however, could be even higher than demonstrated due to loss of material during the recovery process. Although the infective dose (number of oocysts) responsible for the development of infection in humans is unknown, the results indicate that transmission of the parasite to Chinese citizens by consumption of such vegetables raw and unwashed should be taken into consideration. A similar study performed in Poland showed that number of *T. gondii* oocysts present in vegetable samples collected from open markets and greengrocers was less than ten^28^.

Although, the morphological integrity, viability, biological activity or virulence of *Toxoplasma* isolates was not investigated in this study, and consequently their ability to infect humans and/or animals remains unknown, the detection of *T. gondii* DNA and evaluation the probable intensity of contamination of with *T. gondii* oocysts in vegetable samples is clear evidence for the presence of the parasite in the food chain and indicates a potential risk for humans.

Consumption of unwashed vegetables and fruits contaminated with *Toxoplasma* has been ranked amongst the highest risk factors for toxoplasmosis in Norway, due to the regularity these are consumed^70^. In parts of China, poor eating habits like neglect of hand washing before meal or dinner preparation has been indicated as a risk factor of toxoplasmosis infection^67^. In areas with high risks for public health, however, where traditional communal ties have not been ruptured, society, through its historical experience, has adopted countermeasures to alleviate such problems. In the case of Xining City, the general contamination background in QTP regarding parasites, which includes other dangerous parasites such as *Echinococcus* spp. and other Taeniidae, has forced the society to adapt by customizing its habits accordingly, like boiling water and vegetables before consumption. This is also a most probable reason why QTP and China in general, displays lower toxoplasmosis levels than the world average. Nevertheless, societal changes, like urbanization or import of different customs, like society in QTP is experiencing now, may rupture such ties, and this is when awareness and education helps in the prevention of threats that were historically managed, and general improvement of public health. Hence, protection against toxoplasmosis and other public health threats can be achieved by adoption of hygiene measures and practices in open markets, as well as sanitary regulations and best practices for larger units and centralised distribution centres, like warehouses and supermarkets.

Considering the possibility of working with contaminated vegetables and fruit as a risk factor of toxoplasmosis, a comparison with the meat industry can be performed. A study in Mexico has concluded that toxoplasmosis is not associated with handling unwashed and raw vegetables in areas with relatively low prevalence of *Toxoplasma* like in China^79^. Therefore, we can assume that the relatively low prevalence rate in vegetables and the occupational characteristics, regarding the handling of vegetables, do not constitute an additional risk for the workers in this sector, in contrast with other agro-industries like the meat producing. This is in contrast with the meat processing and dairy industry, where *Toxoplasma* has been established as a potential occupational risk factor^80–82^, accounting for the differences during the production processes and the conditions workers are exposed. However, more data, from different provinces of China, focused on the presence of *Toxoplasm*a in vegetables is needed to draw unequivocal conclusions.

## Conclusions

Toxoplasmosis is a persistent problem in China. In this study, we report contamination of vegetable samples available in local open market in Xining City in the Qinghai-Tibet Plateau, in China, with *T. gondii*. The detection of *T. gondii* DNA in vegetable samples is evidence of the presence of the parasite in the agricultural environment and its persistence through multiple stages in the food sector, indicating a potential risk for humans. Therefore, results of our study call for more concentrated screening of the environmental matrices, soil, vegetables and the different production stage to establish the transmission routes, and the level of risk to contract toxoplasmosis through contact with contaminated vegetables. Furthermore, revision and adoption of food safety practices in order to prevent *T. gondii* from reaching the consumers through the food chain, in this and other parts of China.

## Methods

### Recovery and concentration of oocysts from vegetable samples

At first vegetables were rinsed thoroughly. In order to concentrate and recover *Toxoplasma gondii* oocysts from washing suspensions, Al_2_(SO_4_)_3_ flocculation methodology was employed^83^. Finally, the suspension obtained was preserved at −20 °C for further analysis.

### DNA extraction

Prior to DNA extraction, the material obtained from vegetable samples was prepared using ten freeze-thaw cycles (using liquid nitrogen and a water bath) to destroy the walls of the oocysts and improve the efficiency of DNA extraction. Afterwards, DNA extraction was performed using a commercial TIANamp Micro DNA Kit (DP 316) (Tiangen Biotech, Beijing, China) according to the manufacturer’s instructions. The DNA was then stored at −20 °C.

### Specific detection of *T. gondii* DNA by real-time PCR

For specific detection of *Toxoplasma gondii* DNA real-time PCR assay based on *T. gondii* B1 gene^84^ was used. Each vegetable sample was examined three times. Briefly, the amplification reaction mixture consisted of 12.5 μL of Real-Time 2x HS-PCR Master Mix Probe (A&A Biotechnology, Gdynia, Poland), 400 nM of each primer (Genewiz SZ, Suzhou, China), 80 nM of TaqMan probe (Genewiz SZ, Suzhou, China), and 5 μL of template DNA in a 25 μL reaction volume. Amplification was performed with an initial polymerase activation step (10 min at 95 °C), followed by 40 cycles of denaturation (15 s at 95 °C) and hybridisation/extension (1 min at 60 °C) in a AriaMx Real-time PCR thermocycler, Agilent). PCR products were analysed using AriaMx Real-time PCR System Software. The cycle threshold (CT) value, determining the cycle number at which the reporter’s fluorescence exceeds the threshold value, was recorded. A sample was considered positive if the CT value was < 40.

All PCR experiments were performed including *T. gondii* positive controls to ensure the correct functionality of the reaction, as well as negative controls to ensure that no PCR component had been contaminated. The DNA isolated from tachyzoites of parasite (the *Toxoplasma gondii* RH strain), obtained from the National Institute of Hygiene, Poland was used as positive control in the performed experiments.

Additionally, samples were retested for the presence of PCR inhibitors by mixing 4 μL of DNA template and 1 μL of internal positive control (IPC) (more details concerning the IPC used are available in Supplementary information). Comparison of results of amplification (Cq values) obtained for samples containing combination of IPC and template DNA with IPC alone allowed estimation of potential interference.

### Sequencing

The final PCR products from the positive samples were sequenced. Both forward and reverse orientation cycle sequencing was performed using the amplification primers. The sequences obtained were then analysed using GeneStudio^TM^ Professional (GeneStudio, Inc., USA) and ChromasDNA sequencing software.

### Genotyping

*T. gondii* genotypes were determined using multilocus PCR–RFLP assay with selected genetic markers: SAG1, SAG2, SAG3, BTUB, GRA6, c22-8, c29-2, L358, PK1 and Apico^19,85,86^. The set of reaction included nested PCR and restriction analysis of amplified products.

#### Amplification of genetic markers using nested PCR

The 1^st^ step of nested PCR was performed using set of external primers (Table 3) in a 25 μL reaction volume and the amplification reaction mixture consisted of 12.5 μL of the standard and ready-to-use PCR mixture 2xPCR Mix Plus High GC (A&A Biotechnology, Poland) containing recombinant Taq polymerase, PCR buffer, magnesium chloride, nucleotides, stabilisers, and gel loading buffer, 200 nM of each forward and reverse primer (Genewiz SZ, Suzhou, China), and 2 μL of template DNA. Amplifications were performed with an initial polymerase activation step (5 min at 95 °C), followed by 35 cycles of denaturation (30 s at 94 °C), primers annealing (1 min at 55 °C), strand extension (2 min s at 72 °C), and final extension (7 min at 72 °C). The 2^nd^ step of nested PCR reactions were performed using internal set of primers (Table 3) under following amplification reaction mixture conditions: 12.5 μL of the standard and ready-to-use PCR mixture 2xPCR Mix Plus High GC (A&A Biotechnology, Poland), 400 nM of each primer (Genewiz SZ, Suzhou, China), and 2 μL of template DNA in a 25-μL reaction volume. Amplifications were performed according to the same protocol as in the first reaction, with the exception that annealing temperature was 60 °C.

**Table 3:** Summary of primers, enzymes and conditions used for multiplex multilocus nested PCR-RFLP typing of *T. gondii* isolates.

Marker		PCR primers (5′-3′)	Size (bp)	Restriction enzymes	Enzyme digestion conditions
SAG1^87^	SAG1 Ex F: SAG1 Ex R: SAG1 In F: SAG1 In R:	GTTCTAACCACGCACCCTGAG AAGAGTGGGAGGCTCTGTGA CAATGTGCACCTGTAGGAAGC GTGGTTCTCCGTCGGTGTGAG	390	Cfr13I + BfoI, Thermo Scientific (double digest)	37 °C 1 h
SAG2^86^	SAG2 Ex F: SAG2 Ex R: SAG2 In F: SAG2 In R:	GGAACGCGAACAATGAGTTT GCACTGTTGTCCAGGGTTTT ACCCATCTGCGAAGAAAACG ATTTCGACCAGCGGGAGCAC	546	HinfI + TaqI, Thermo scientific	37 °C 1 h + 65 °C 1 h
SAG3^87^	SAG3 Ex F: SAG3 Ex R: SAG3 In F: SAG3 In R:	CAACTCTCACCATTCCACCC GCGCGTTGTTAGACAAGACA TCTTGTCGGGTGTTCACTCA CACAAGGAGACCGAGAAGGA	311	BcnI, Thermo Scientific	37 °C 1 h
c 22-8^86^	C22-8 Ex F: C22-8 Ex R: C22-8 In F: C22-8 In R:	TGATGCATCCATGCGTTTAT CCTCCACTTCTTCGGTCTCA TCTCTCTACGTGGACGCC AGGTGCTTGGATATTCGC	521	Alw26I + MboII, Thermo Scientific (double digest)	37 °C 1 h + 55°C 1 h
c 22-9^86^	C29-2 Ex F: C29-2 Ex R: C29-2 In F: C29-2 In R:	ACCCACTGAGCGAAAAGAAA AGGGTCTCTTGCGCATACAT AGTTCTGCAGAGTGTCGC TGTCTAGGAAAGAGGCGC	446	TaiI + RsaI, Thermo Scientific (double digest)	37 °C 1 h
L358^86^	L358 Ex F: L358 Ex R: L358 In F: L358 In R:	TCTCTCGACTTCGCCTCTTC GCAATTTCCTCGAAGACAGG AGGAGGCGTAGCGCAAGT CCCTCTGGCTGCAGTGCT	418	BsuRI + Hin1II, Thermo Scientific (double digest)	37 °C 1 h
BTUB^86^	BTUB Ex F: BTUB Ex R: BTUB In F: BTUB In R:	TCCAAAATGAGAGAAATCGT AAATTGAAATGACGGAAGAA GAGGTCATCTCGGACGAACA TTGTAGGAACACCCGGACGC	411	Bsh1285I + TaqI, Thermo Scientific (double digest)	65 °C 1 h
PK1^86^	L358 Ex F: L358 Ex R: L358 In F: L358 In R:	TCTCTCGACTTCGCCTCTTC GCAATTTCCTCGAAGACAGG AGGAGGCGTAGCGCAAGT CCCTCTGGCTGCAGTGCT	903	Eco88I + RsaI, Thermo Scientific (double digest)	37 °C 1 h
GRA6^86^	GRA6 Ex F: GRAG Ex R: GRA6 In F: GRA 6 In R:	ATTTGTGTTTCCGAGCAGGT GCACCTTCGCTTGTGGTT TTTCCGAGCAGGTGACCT TCGCCGAAGAGTTGACATAG	344	Tru1I, Thermo Scientific	37 °C 1 h
Apico^86^	APICO Ex F: APICO Ex R: APICO In F: APICO In R:	TGGTTTTAACCCTAGATTGTGG AAACGGAATTAATGAGATTTGAA GCAAATTCTTGAATTCTCAGTT GGGATTCGAACCCTTGATA	640	AflII + DdeI, Thermo Scientific (double digest)	37 °C 1 h

#### Restriction analysis of nested PCR products

In order to perform restriction analysis 10 µL of PCR product were mixed with 2 µL of 10x digestive buffer and 1 U of restriction enzyme (each of the two restriction enzymes in case of double digestion) (Thermo Scientific, USA). The reaction was carried out according to manufacturer’s instruction. RFLP products were analysed using a WD-9413B gel imaging analysis system (Beijing Liuyi Biotechnology, China) following electrophoresis on a 3% gel agarose (Biowest Regular Agarose G-10, Gene Company) stained with ExRed nucleic acid electrophoresis dye (Beijing Zoman Biotechnology, China).

### Determination of initial copy numbers of *T. gondii* DNA using qPCR

**Figure 3:**
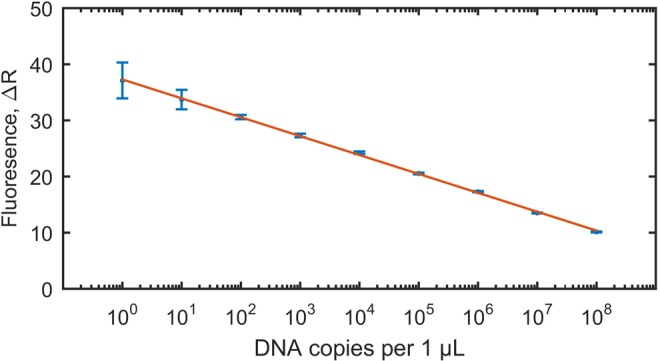
Standard curve, generated based on amplification of the three series of eight dilutions of standard DNA in the range from one to 10^8^ DNA copies per one μL, with marked standard deviations of Cq(ΔR) values obtained for standard DNA triplicates. Efficiency E [%] = 98; Slope, S = −3.37; Y intercept y_int_ = 37.32, Coefficient of determination, R^2^ = 0.99. The standard curve served for quantification of initial copy numbers of *T. gondii* B1 gene in investigated vegetable samples collected from the open markets located in Xining City, Qinghai Province, P.R. China.

In order to determine the initial copy number of the detected *T. gondii* DNA, each positive sample was tested with qPCR based on a standard curve. The standard template was generated by cloning the insert gene (a fragment of the B1 gene amplified by PCR using the set of primers described above) into a plasmid. To generate the standard curve, three series of nine dilutions of standard DNA in the range from one to 10^8^ DNA copies per one μL were prepared (Fig. 3). Following amplification of the standard dilution series, the standard curve was obtained by plotting the log of the initial template copy number against the CT value generated from each dilution. Amplification of the standard dilution series and of the DNA isolated from vegetable samples was run on the same plate. Comparing the CT values of the unknown samples with the standard curve thus enabled the quantification of initial copy numbers.

Initial copy numbers of *T. gondii* DNA were used to calculate equivalent of *T. gondii* dispersive forms that might be present in the examined samples after flocculation (recovery procedure). According to our previous experiments, using real-time PCR, it is possible to detect single *T. gondii* oocyst in water suspension^13^. Thus, the number of *T. gondii* oocysts (oocyst charge of the sample) was estimated using following formula: ${\rm{OCS}}=\frac{{\rm{N}}}{35}\times \frac{1}{A}\times {\rm{B}}$ where: OCS is the oocyst charge of the sample; N is the initial copy number determined using qPCR (per amount of DNA template taken for PCR); 35 is the number of copies of the B1 gene per one *T. gondii* cell; *A* = 8, the number of *T. gondii* sporozoites per one oocyst; B is the multiplication factor referring to a total amount of DNA extracted from investigated sample.

### Statistical analysis

In order to investigate the correlation between *T. gondii* presence in investigated vegetable samples and sampling period statistical analysis was performed using the Pearson chi-square test (χ^2^). Calculations were performed using MATLAB 2015a.

## Supplementary information

Supplementary Information

## Data Availability

All data generated or analysed during this study are included in this published article.
