# Supplementary material for: First molecular detection of Toxoplasma gondii in vegetable samples in China using qualitative, quantitative real-time PCR and multilocus genotyping
Source: Sci Rep. 2019 Nov 26;9:17581. doi: 10.1038/s41598-019-54073-6 (PMC6879479; doi:10.1038/s41598-019-54073-6)
Supplement: Supplementary file 1 — Supplementary Information [file 41598_2019_54073_MOESM1_ESM.pdf]

Supplementary Information

First molecular detection of *Toxoplasma gondii* in vegetable samples in China using qualitative, quantitative real-time PCR and multilocus genotyping.

Anna Lass[1,2*], Liqing Ma[2], Ioannis Kontogeorgos[2,3] Xueyong Zhang[2], Xiuping Li[2], Panagiotis Karanis[2,4 ,5]

[1]Department of Tropical Parasitology, Institute of Maritime and Tropical Medicine in Gdynia, Medical University of Gdansk, 9b Powstania Styczniowego Str., 81-519 Gdynia, Poland anna.lass@gumed.edu.pl

[2]State Key Laboratory of Plateau Ecology and Agriculture, Center for Biomedicine and Infectious Disease, Qinghai University, 1#Wei'er Road, Qinghai Biological Scientific Estate Garden, Xining 810016 - P. R. China, maliq67@hotmail.com, zhang_xyong@163.com, 2216659903@QQ.com

[3]Marine Sciences Department, School of Environment, University of the Aegean, University Hill, 88 100 Mytilene, Greece, ikontogeorgos@isc.tuc.gr

[4]Cologne University, Medical Faculty and University Hospital, Cologne, Germany, panagiotis.karanis@uk-koeln.de

[5]University of Nicosia, Medical School, 46 Makedonitissas Avenue, CY-2417 P.O. Box 24005, CY-1700, Nicosia, Cyprus

*Corresponding author: Anna Lass, Department of Tropical Parasitology, Institute of Maritime and Tropical Medicine in Gdynia, Medical University of Gdansk, 9b Powstania Styczniowego Str., 81-519 Gdynia, Poland anna.lass@gumed.edu.pl

Supplementary Table S1. Characteristics of standard curve generated by amplification of the serial dilutions of standard DNA.

Standard template No	NS	Cq(ΔR) Set1	Cq(ΔR) Set2	Cq(ΔR) Set3	Cq(ΔR) av.	Cq(ΔR) SD
**1**	1.00e+008	10.02	10.15	10.20	10.13	0.09
**2**	1.00e+007	13.50	13.58	13.42	13.50	0.08
**3**	1.00e+006	17.18	17.39	17.34	17.30	0.11
**4**	1.00e+005	20.44	20.45	20.72	20.54	0.16
**5**	1.00e+004	23.99	24.32	24.42	24.24	0.23
**6**	1.00e+003	27.60	27.38	26.96	27.31	0.33
**7**	1.00e+002	30.15	30.84	30.80	30.60	0.39
**8**	1.00e+001	32.32	33.17	35.66	33.72	1.74
**9**	1.00e+000	39.38	34.87	–	37.12	3.19
**Characteristics of the standard curve (produced by Agilent Aria Software v1.3)**						
**E [%]** = 98, $\mathbf{R^2}$ = 0.99, **S** = -3.37, $\mathbf{y_{int}}$ = 37.32						

NS – initial concentration of standard DNA [copies/µL]
Cq (ΔR) set1-3 – Cq values obtained for particular set of replicates
Cq (ΔR) av. – average Cq values obtained from three sets of replicates
Cq (ΔR) SD – standard deviation obtained from three sets of replicates
E – the amplification efficiency
R^2 – the correlation coefficient
S – slope of the standard curve
y_{int} – the y-intercept for log(N) = 0

Supplementary Table S2. Summary result of *T. gondii* qPCR performed for vegetable samples collected from open markets in the Xining City, Qinghai Province, P.R. China.

Positive sample No	Cq(ΔR) 1	Cq(ΔR) 2	Cq(ΔR) 3	Cq(ΔR) av.	Cq(ΔR) SD	NPav.	OCSav.	SCSav.	CCSav.
**32**	31.55	31.68	31.60	31.61	0.06	4.94e+001	1.7	3.4	13.6
**50**	27.90	27.72	27.59	27.74	0.15	6.99e+002	25	50	200
**61**	17.50	17.41	17.50	17.47	0.05	7.74e+005	27640	55280	221120
**140**	20.59	20.47	20.53	20.53	0.06	9.56e+004	340	683	2730
**192**	34.73	34.17	33.4	34.10	0.66	9.66e+000	0.3	0.69	2.76
**203**	27.67	27.78	27.74	27.73	0.06	7.00e+002	25	50	200
**212**	17.41	17.47	17.65	17.51	0.12	7.56e+005	27000	54000	432000
**229**	31.21	30.80	31.65	31.22	0.42	6.64e+001	2.4	4.8	19.2
**254**	30.55	30.44	29.90	30.23	0.29	1.29e+002	4.6	9.2	36.8
**265**	33.93	–	38.43	36.18	3.18	5.30e+000	0.18	0.36	1.44

Cq (ΔR) 1-3 – Cq values obtained for positive vegetable samples in qPCR run in triplicate together with standard curve
Cq (ΔR) av. – average Cq values obtained from triplicates of positive vegetable samples
Cq (ΔR) SD – standard deviation obtained from triplicates of positive vegetable samples
NP – initial copy number (number of copies of *T. gondii* B1 gene) present in positive vegetable samples calculated based on standard curve
OCS – calculated equivalent of initial number of *T. gondii* oocysts present in positive vegetable samples
SCS – calculated equivalent of initial number of *T. gondii* sporocysts present in positive vegetable samples (OCS x 2, one oocyst consist of two sporocysts)
CCS – calculated equivalent of initial number of *T. gondii* cells (sporozoites) present in positive vegetable samples (OCS x 8, one oocysts consist of two sporocysts filled with four sporozoites each)

Supplementary Table S3. Results of inhibition test performed for *Toxoplasma gondii* positive vegetable samples collected from open markets located in Xining City, Qinghai Province, P.R. China.

Number of template	Cq (ΔR) for IPC
**32**	15.12
**50**	15.37
**61**	15.15
**140**	15.32
**192**	15.09
**203**	15.58
**212**	15.69
**229**	15.32
**254**	15.39
**265**	15.26

IPC – internal positive control
Cq (ΔR) IPC - Cq values obtained after amplification of internal positive control (IPC)

Supplementary data for internal positive control (IPC) used in the study

DNA extracted from laboratory strain of *Acanthamoeba* spp. cultivated axenically in the laboratory of the Department of Tropical Parasitology, Medical University of Gdansk was used to construct IPC. Briefly, a 180 bp fragment of 18SrRNA gene of *Acanthamoeba* spp. was amplified using primers AcantF900 and AcantR1100 [1] and cloned into a plasmid. Detection of IPC was performed with real-time PCR using TaqMan probe labelled with 5' Cy5/3'BHQ3 fluorophores under amplification conditions described in the manuscript.

Bibliography

1. Qvarnstrom, Y., Visvesvara, G. S., Sriram, R. & Silva, A. J. da. Multiplex Real-Time PCR assay for simultaneous detection of *Acanthamoeba* spp., *Balamuthia mandrillaris*, and *Naegleria fowleri*. *Journal of Clinical Microbiology* **44**, 3589–3595 (2006).
